# Effect of Biofunctional Green Synthesized MgO-Nanoparticles on Oxidative-Stress-Induced Tissue Damage and Thrombosis

**DOI:** 10.3390/molecules27165162

**Published:** 2022-08-12

**Authors:** Manjula M. Venkatappa, Chikkappa Udagani, Sujatha M. Hanumegowda, Siddanakoppalu N. Pramod, Shivakumar Venkataramaiah, Rajesh Rangappa, Rajeshwara Achur, Abed Alataway, Ahmed Z. Dewidar, Mohamed Al-Yafrsi, Eman A. Mahmoud, Hosam O. Elansary, Devaraja Sannaningaiah

**Affiliations:** 1 Department of Biochemistry, Kuvempu University, Shankaraghatta, Shimoga 577451, India; 2 Department of Physics, University College of Science, Tumkur University, Tumkur 572103, India; 3 Department of Food Technology, Davangere University, Shivagangotri, Davanagere 577007, India; 4 Centre for Bioscience and Innovation, Department of Studies and Research in Biochemistry, Tumkur University, Tumkur 572103, India; 5 Chromed Bioscience, Tumkur 572106, India; 6 Prince Sultan Bin Abdulaziz International Prize for Water Chair, Prince Sultan Institute for Environmental, Water and Desert Research, King Saud University, Riyadh 11451, Saudi Arabia; 7 Department of Agricultural Engineering, College of Food and Agriculture Sciences, King Saud University, Riyadh 11451, Saudi Arabia; 8 Plant Production Department, College of Food & Agriculture Sciences, King Saud University, Riyadh 11451, Saudi Arabia; 9 Department of Food Industries, Faculty of Agriculture, Damietta University, Damietta 34511, Egypt

**Keywords:** MgO NPs, *Tarenna asiatica*, TAFE, antioxidant, anti-coagulant, anti-platelet

## Abstract

The present study describes the green biofunctional synthesis of magnesium oxide (MgO) nanoparticles using the aqueous *Tarenna asiatica* fruit extract. The characterization of *Tarenna asiatica* fruit extract MgO nanoparticles (TAFEMgO NPs) was achieved by X-ray powder diffraction, UV-Vis spectroscopy, FTIR, TEM, SEM, and energy-dispersive X-ray diffraction. TAFEMgO NPs scavenged the DPPH free radicals with an IC50 value of 55.95 μg/μL, and it was highly significant compared to the standard. To authenticate the observed antioxidant potential of TAFEMgO NPs, oxidative stress was induced in red blood cells (RBC) using sodium nitrite (NaNO_2_). Interestingly, TAFEMgO NPs ameliorated the RBC damage from oxidative stress by significantly restoring the stress parameters, such as the protein carbonyl content (PCC), lipid peroxidation (LPO), total thiol (TT), super-oxide dismutase (SOD), and catalase (CAT). Furthermore, oxidative stress was induced in-vivo in Sprague Dawley female rats using diclofenac (DFC). TAFEMgO NPs normalized the stress parameters in-vivo and minimized the oxidative damage in tissues. Most importantly, TAFEMgO NPs restored the function and architecture of the damaged livers, kidneys, and small intestines by regulating biochemical parameters. TAFEMgO NPs exhibited an anticoagulant effect by increasing the clotting time from 193 s in the control to 885 s in the platelet rich plasma. TAFEMgO NPs prolonged the formation of the clot process in the activated partial thromboplastin time and the prothrombin time, suggest the effective involvement in both intrinsic and extrinsic clotting pathways of the blood coagulation cascade. TAFEMgO NPs inhibited adenosine di-phosphate (ADP)-induced platelet aggregation. TAFEMgO NPs did not show hemolytic, hemorrhagic, and edema-inducing properties at the tested concentration of 100 mg/kgbody weight, suggesting its non-toxic property. In conclusion, TAFEMgO NPs mitigates the sodium nitrite (NaNO_2_)- and diclofenac (DFC)-induced stress due to oxidative damage in both in vitro and in vivo experimental models.

## 1. Introduction

Uncontrolled biological oxidation in the human body generates free radicals; at lower concentrations (1–100 nM), they kill the pathogens and regulate the cell growth [1]. However, at a higher rate (above 100 nM), they modify lipids, proteins, and DNA and damage vital tissues. Thus, oxidative stress is considered to be the modifier in the pathogenesis of thrombosis, hypertension, cancer, cardiovascular diseases, atherosclerosis, and diabetes mellitus. Most importantly, oxidative stress elicits erythrocyte damage (eryptosis), and the intern generates RBC-derived ROS (RBC-ROS) that plays a predominant role in the pathogenesis of the said diseases. Perhaps, RBC-ROS-induced anemia, hypoxia, cardiovascular diseases, tissue destruction, and thrombosis are more prevalent [2]. Thus, oxidative stress is a burning issue of this era. Several research groups documented the antioxidant utility in the management of stress-related complications from natural sources [3]. Yet, nanotechnology has emerged as a potential tool due to its immense therapeutic applications, such as anti-inflammatory, antibacterial, anticancer, antioxidant, antitumor, drug delivery, and bio-absorption [4,5,6].

Nanoparticles are synthesized using physical, chemical, and biological approaches (laser vaporization, wet impregnation, ultra-sonication, green route method, and precipitation methods) [7]. Over the past decades, metal oxide nanoparticles (TiO_2_, NiO, ZnO, MgO, and CuO) have been characterized based on their unique intrinsic functionalities and thermal/magnetic features and were found to exhort biological toxicity. However, metal oxide nanoparticles synthesized using green technology showed the least biological toxicity with immense antioxidant, anti-inflammatory, antimicrobial, and anticancer properties [8,9]. Thus, the biofunctionalized nanoparticles through green synthesis from natural resources (plants and microbes) receive high attention because of ecofriendly, stable, low cost production, high bioavailability, and non-toxic nature [10,11]. The bioactive transformation of green synthesized metal oxide nanoparticles could be due to the plethora of phytoconstituents stored in the plants (proteins, amino acids, vitamins, alkaloids, flavonoids, polyphenols, tannins, terpenoids, and alcoholic compounds) that act as the stabilizing/reducing agent or the controlled precipitation, enriching the therapeutic potency [12,13].

*Tarenna asiatica* belongs to the plant family, *Rubiaceae,* and was reported to be a native to the Asian continent. According to the literature survey, various parts of *Tarenna asiatica* have been used in folk medicine to cure several ailments with its anti-inflammatory, antioxidant, anti-hypertensive, and anticancer properties [14,15]. Therefore, in the current study, *Tarenna asiatica* fruit extract was employed for green biosynthesis of MgO bio functional nanoparticles and their protective effect on oxidative stress-induced damage of RBCs, livers, kidneys, and small intestines. Additionally, the antithrombotic activity was examined to elucidate the possible protective and therapeutic potential of the biofunctionalized MgO nanoparticles. Hence, they may be the better candidates to treat the pathogenesis of oxidative stress.

## 2. Results

### 2.1. Characterization Studies of MgO NPs

#### 2.1.1. XRD Analysis of TAFEMgO NPs

**Figure 1:**
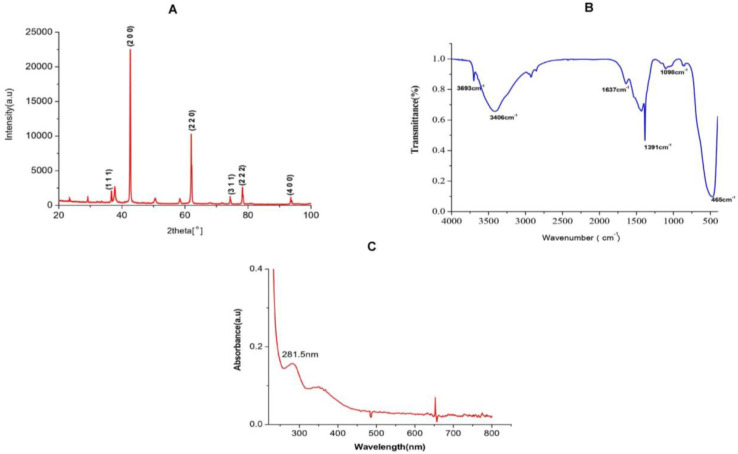
(**A**) XRD detector pattern of TAFEMgO NPs. The high dense peak at 2θ = 42.55° with interplanar spacing (d-spacing) 2.122 Å is due to (2 0 0) reflection of cubic MgO. (**B**) Fourier transform infrared spectroscopy (FTIR) spectrum of TAFEMgO NPs showing stretching band of synthesized TAFEMgO NPs. (**C**) UV-visible spectroscopy pattern of TAFEMgO NPs showing two reflection minima at 210 and 281 nm.

The X-ray diffractions (XRD) were carried out using the XRD diffractometer. The X-ray diffracted grams of TAFEMgO NPs exhibited intense peaks at 2θ angles of 36.71°, 42.55°, 61.955°, 74.37°, 78.234°, and 93.528° (Figure 1A). The noticed XRD peaks were consistent with the standard JCPDS number, 45–946. XRD peaks obtained at 2θ angles 36.71°, 42.55°, 61.955°, 74.37°, 78.234°, and 93.528°are indexed, respectively, for crystal planes (1 1 1), (2 0 0), (2 2 0), (3 1 1), (2 2 2), and (4 0 0) of the F -m -3 m space group, cubic MgO with lattice constant a = 4.2112 Å. The most intense peak at 2θ = 42.55° with interplanar spacing (d-spacing) 2.122 is due to (2 0 0) reflection of cubic MgO. The size of the crystallite of synthesized NPs were determined by employing the standard Debye Scherer equation: D = (K × λ)/βCos, where the K value was 0.94, and it represents the shape factor for spherical crystallites having cubic symmetry; λ is 1.584 A); β considered as FWHM (Full Width Half Maximum in peak radians); and θ is the known Bragg’s angle. The major peak in XRD corresponds to reflection plane (2 0 0) was chosen to calculate the average crystalline size. The β of the major reflection plane (2 0 0) was found to be 0.2187°, and the average NPs crystallite size was 40.74 nm.

#### 2.1.2. FTIR Analysis of TAFEMgO NPs

The FTIR analytical technique is used for the identification of active functional reactive groups representing test sample at the wave number 400–4500 cm^−1^. The bioactive compounds in the aqueous extract of *Terenna asiatica* fruit extract (TAFE) were responsible for controlled precipitation leading to the MgO NPs, which were assessed for modification using the FTIR technique. The FTIR profile of the TAFEMgO NPs showed six major characteristic bands, suggesting the presence of diversified organic functional groups (Figure 1B). The observed band at 3406 cm^−1^ signifies the stretch of -OH group. The band at 1637 cm^−1^ supports the presence of (C=O) NH (amide group). The bending peak representing at 1396 cm^−1^ corresponds to vibration of -OH groups and the peak observed at 485 cm^−1^ was corresponds to vibration due to stretching of MgO. The peak at 3693 cm^−1^ resulted from anti-symmetric stretching vibrations in Mg(OH)_2_ crystallite structure.

#### 2.1.3. UV-Vis Spectral Analysis of TAFEMgO NPs

The UV-Vis absorbance study is a versatile technique that helps with the preliminary characterization of synthesized nanoparticles along with the molecular species present in them. Thus, TAFEMgO NPs were subjected to UV-Vis spectroscopy. Interestingly, TAFEMgO NPs showed a UV absorption peak at 281.5 nm (4.41 eV) to 350 nm (4.8 eV). Figure 1C represents the UV spectra of TAFEMgO NPs. The recorded UV-Vis absorption peaks were in agreement with the currently reported literature.

#### 2.1.4. SEM, EDX-ray Diffraction, TEM, and Selective Electron Diffraction (SED) Study of TAFEMgO NPs

**Figure 2:**
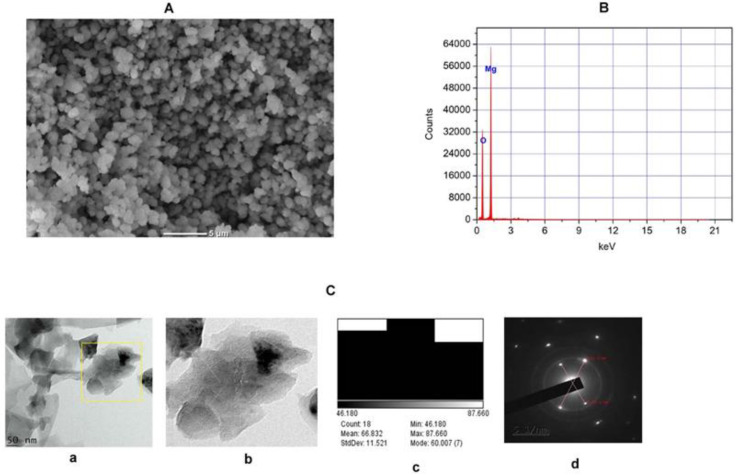
(**A**) SEM microgram of TAFEMgO NPs. The SEM micrograph of TAFEMgO NPs was depicted with scale bar 5 μm. (**B**) ED x-ray diffraction (EDX) reveals high elemental composition with Mg and O, showing high purity with mass% of 33.70 and 66.90, respectively, and 1.253 and 0.525 are the keV of TAFEMgO NPs, respectively, confirm the formation of MgO NPs. (**C**) Transmission electron microscope (TEM): (**Ca**) TEM micrograph of MgO, (**Cb**) enlarged view, (**Cc**) particle size distribution, (**Cd**) selected area electron diffraction (SAED) for MgO. Average particle size estimated for TAFEMgO NPs is 66.832 nm.

SEM (Scanning Electron Microscopy) was utilized to characterize the appearance of grain shaped MgO nanoparticles. Due to the poor resolution of the SEM, the shapes of the TAFEMgO NPs were not clear. However, the data obtained from SEM revealed that the TAFEMgO NPs were nearly spherical/polygonal shaped with uniform distribution (Figure 2A). EDX was performed to characterize the elemental composition percentage of TAFEMgO NPs. The EDX spectrum of TAFEMgO NPs revealed the presence of energy peaks of magnesium and oxygen at 1.253 keV and 0.525 keV, respectively (Figure 2B). The mass percentages of Mg and O were found to be 33.70 and 66.90, respectively. Further, MgO nanoparticles were characterized from the TEM using the open-source software, Image J. The TEM (Transmission Electron Microscopy) data suggests the TAFEMgO NPs were nearly spherical /polygonal in shape, having nanoparticles with average sizes of 66.832 nm. Figure 2(Ca) represents the TEM micrograph of the TAFEMgO NPs with scale bar of 50 nm, and Figure 2(Cb) is the enlarged view of the TEM micrograph. The size distributions of TAFEMgO NPs was shown in Figure 2(Cc). The selective area electron diffraction (SAED) full technique is used for understanding the crystal structure of the nanoparticles. Interestingly, the SAED image suggests the TAFEMgO NPs were crystalline in nature (Figure 2(Cd)).

### 2.2. TAFEMgO NPs Scavenge Free Radicals In Vitro

**Figure 3:**
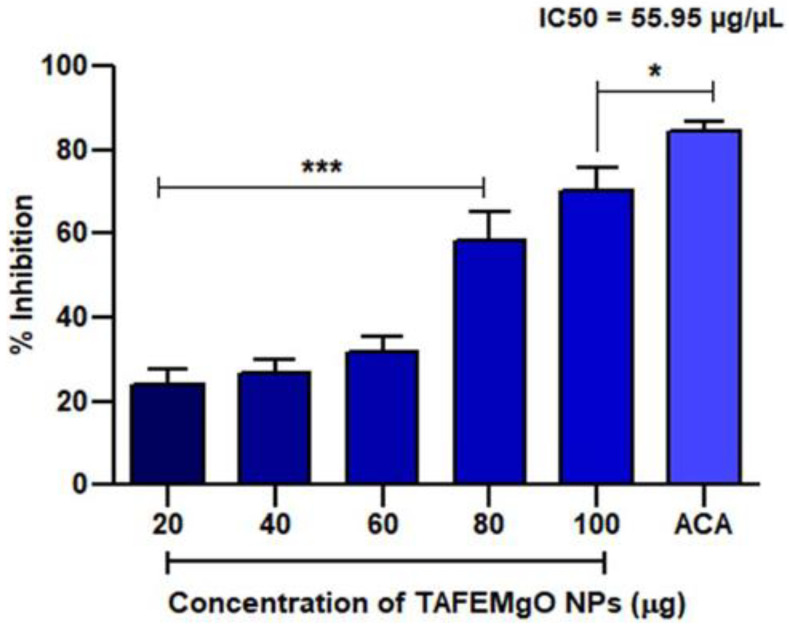
TAFEMgO NPs DPPH-scavenging activity. The DPPH technique was employed to predict the antioxidant potential of TAFEMgO NPs. Each value is given as a mean ± SD. * Significance at *p* ≤ 0.005 and *** at *p* ≤ 0.001.

TAFEMgO NPs showed antioxidant properties by scavenging the Di-Phenyl-2-Picryl-Hdrazyl (DPPH) free radical in vitro through concentration-dependent manner. About 70% of antioxidant potential was noticed against ascorbic acid, a positive control, and it showed 55.95 μg of mean IC_50_ value. Figure 3 represents the antioxidant activity of TAFEMgO NPs.

### 2.3. TAFEMgO NPs Ameliorate NaNO_2_-Induced Stress Markers in the RBC Model (In Vitro)

**Figure 4:**
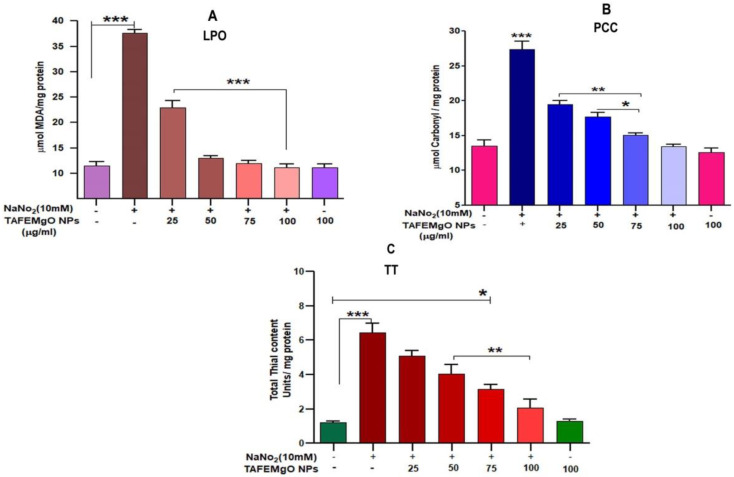
Effect of TAFEMgO NPs on NaNO_2_-induced oxidative stress in RBCs: (**A**) lipid peroxidation, (**B**) protein carbonyls (**C**) total thiols. To determine the mentioned activities of TAFEMgO NPs against NaNO_2_-induced oxidative damage, RBCs were pre-incubated for 10 min with various doses (25–100 g/mL) of TAFEMgO NPs at 37 °C prior to treatment with NaNO_2_ (10 mM). The results were presented in average units/mg of protein and were expressed as mean ± SEM (*n* = 3). * Significance at *p* ≤ 0.005, ** at *p* ≤ 0.001 and *** at *p* ≤ 0.0001.

The antioxidant role of TAFEMgO NPs was further authenticated using NaNO_2_-induced oxidative stress in the RBC model. Major biomarkers for oxidative stress, such as LPO, PCC, and TT levels in the treated RBC samples were examined. The amount of lipid peroxidation (LPO) was expressed in terms of the level of malondialdehyde. The samples treated with NaNO_2_-the level of malondialdehyde (MDA) was elevated significantly, while TAFEMgO NPs normalized the MDA level in a dose-dependent pattern significantly (*p* < 0.001). Figure 4A represents the causative protective property of TAFEMgO NPs on NaNO_2_-induced lipid peroxidation (LPO). Further, there was an observed elevation in the content of protein carbonyl (PC) in the NaNO_2_-treated RBC. Whereas, in the case of the TAFEMgO NPs pre-incubated with RBC, the level of PCC was significantly restored in comparison with control sample (Figure 4B). Similarly, NaNO_2_-treated RBC represents an elevated level of the total thiol content. For TAFEMgO NPs pre-treated with RBC, the level of total thiol content was restored to normal compared to the control (Figure 4C). Interestingly, for TAFEMgO NPs treated alone with RBC, all three parameters were unaltered compared to the control RBC. Further, NaNO_2_-treated RBCs indicated considerable decrease in antioxidant enzymes (SOD and CAT) activity (Supplementary Materials, Figures S1 and S2), whereas, TAFEMgO NPs (0–100 μg) pre-treated with RBCs showed significance (*p* < 0.0001); and restored the level of the SOD-and-CAT-treated RBC sample.

### 2.4. TAFEMgO NPs Ameliorate the Diclofenac-Induced Oxidative Stress Markers (In Vivo)

**Figure 5:**
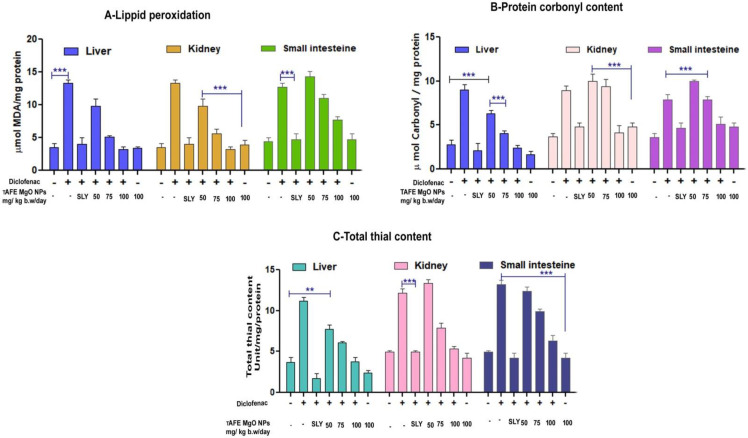
Effect of TAFEMgO NPs on oxidative stress induced by diclofenac in small intestines, kidneys, and livers: (**A**) lipid peroxidation, (**B**) protein carbonyls (**C**) total thiols and control, diclofenac (50 mg/kg), SLY + diclofenac (25 mg/kg), TAFEMgO NPs (50 mg/kg) + diclofenac, TAFEMgO NPs (75 mg/kg) + diclofenac, TAFEMgO NPs (100 mg/kg) + diclofenac, and TAFEMgO NPs alone (100 mg/kg) in comparison to the toxicity for control group. All samples presented were administered as mg/kg bodyweight/day. The data were represented as mean (*n* = 3) ± SEM. ** Significance at *p* ≤ 0.001 and *** at *p* ≤ 0.0001.

In the case of diclofenac-injected (50 mg/kg) rat’s kidney, liver and small intestine homogenates, the levels of PCC, MDA, and TT were elevated significantly in comparison to the positive control silymarin. The TAFEMgO NPs-injected (100 mg/kg) rat’s liver, kidney, and small intestine homogenates displayed significant decreases in the MDA, PCC, and TT levels compared to the normal groups. However, in the TAFEMgO NPs-alone-treated rat’s homogenates, the levels of MDA, PCC, and TT were normal and were in comparison with control groups (Figure 5A–C). The endogenous antioxidant enzymes (SOD and CAT) were significantly reduced in the sample homogenates obtained from DFC-treated rats. In the case of DFC pre-incubated with TAFEMgO NPs (100 mg/kg) injected rats, antioxidant enzyme activity was restored in the kidney, liver and small intestine homogenates. Interestingly, the TAFEMgO NPs-alone-treated groups, SOD and CAT activity was unaltered compared to the control groups (Supplementary Materials, Figures S3 and S4).

### 2.5. TAFEMgO NPs Restore the Biochemical Parameters In Vivo

In the case of the DFC-treated rat’s blood samples, the biochemical parameters, such as albumin, globulin, and total protein levels, were decreased. While, alkaline phosphatase, total bilirubin, indirect bilirubin, directs SGOT (Serum glutamate oxaloacetate transaminase), bilirubin, and SGPT (serum glutamate pyruvate transaminase) increased significantly. DFC pre-incubated with the TAFEMgO NPs-injected group of rats, the level of the said biochemical parameters were restored to normal in comparison with the positive control silymarin-treated group of experimental rats (Table 1).

### 2.6. TAFEMgO NPs Restore the Liver, Kidney, and Small Intestine Morphology

**Figure 6:**
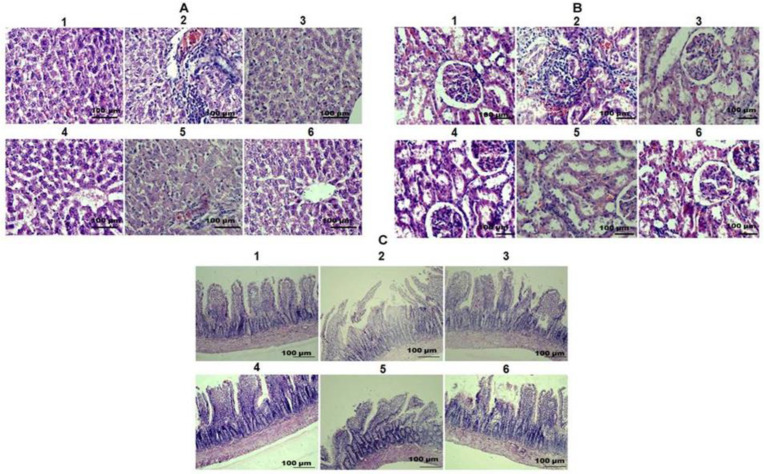
(**A**) Effect of TAFEMgO NPs on histology of liver, kidney, and small intestine: (**A1**) normal liver tissues are shown in the control group; (**A2**) diclofenac-administered (50 mg/kg) group showed vacuolar changes; (**A3**) diclofenac (50 mg/kg) pre-incubated with SLY-administered (25 mg/kg) group showed mild vacuolar changes; (**A4**) diclofenac (50 mg/kg) pre-incubated with TAFEMgO NPs (50 mg/kg) restored normal architecture; (**A5**) diclofenac (50 mg/kg) pre-incubated with TAFEMgO NPs-injected (75 mg/kg) group showed almost normal liver histology; (**A6**) TAFEMgO NPs-alone-injected (100 mg/kg) group showed normal liver histology. (**B**) Effect of TAFEMgO NPs on kidney histopathology: (**B1**) normal kidney tissues are shown in the control group; (**B2**) diclofenac-administered (50 mg/kg) group showed tubular cell necrosis; (**B3**) diclofenac (50 mg/kg) pre-incubated with SLY-injected (25 mg/kg) group showed mild tubular cell necrosis; (**B4**) diclofenac (50 mg/kg) pre-incubated with TAFEMgO NPs (50 mg/kg) restored normal architecture; (**B5**) diclofenac (50 mg/kg) pre-incubated with TAFEMgO NPs-injected (75 mg/kg) group restored almost normal architecture; (**B6**) TAFEMgO NPs-alone-injected (100 mg/kg) group showed normal kidney histology. (**C**) The effect of TAFEMgO NPs on the histopathology of the small intestine: (**C1**) normal small intestine tissues are shown in the control group; (**C2**) diclofenac-injected (50 mg/kg) group showed necrosis and inflammation; (**C3**) diclofenac (50 mg/kg) pre-incubated with SLY-injected (25 mg/kg) group showed mild necrosis and inflammation; (**C4**) diclofenac (50 mg/kg) pre-incubated with TAFEMgO NPs (50 mg/kg) restored normal architecture; (**C5**) diclofenac (50 mg/kg) pre-incubated with TAFEMgO NPs-injected (75 mg/kg) group restored almost normal architecture; (**C6**) TAFEMgO NPs-alone-injected (100 mg/kg) group showed normal small intestine histology. All the test samples were administered to control and test groups as mg/kg bodyweight/day.

Microscopic examination of the livers, kidneys, and small intestines from the control group (Figure 6(A1,B1,C1)) and the TAFEMgO NPs-alone-treated group (Figure 6(A6,B6,C6)) did not show any abnormality of tissue morphology compared to the positive (silymarin) control-treated group of animals (Figure 6(A3,B3,C3)). However, in the diclofenac-treated rats, massive destruction of liver, kidney, and small intestine tissues was indicated (Figure 6(A2,B2,C2)). Hepatocellular degeneration was observed in liver histology through increased cell infiltration. Tubular degeneration was found in the kidneys. Villi necrosis was noticed in the small intestine section. Interestingly, synthesized nanoparticles restored the structures of the damaged livers, kidneys, and small intestines from diclofenac-induced oxidative stress (Figure 6(A4,A5,B4,B5,C4,C5)) in a dose-dependent manner.

### 2.7. TAFEMgO NPs Indicated Anticoagulant and Antiplatelet Properties

**Figure 7:**
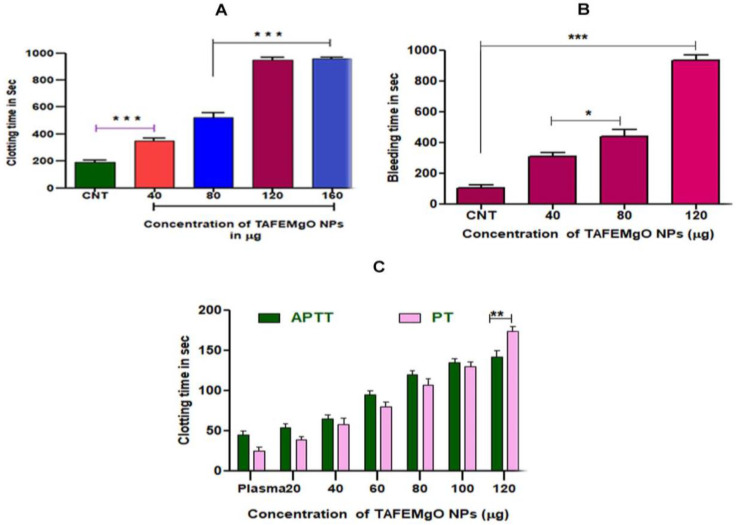
(**A**) Plasma recalcification time of TAFEMgO NPs: CNT-control clotting time was increased with TAFEMgO NPs in a dose-dependent (20–100 μg) manner. (**B**) Tail-bleeding time of MgO NPs: intravenous injection of TAFEMgO NPs to experimental rats caused dose-dependent increase in bleeding time with CNT (control). (**C**) Concentration-dependent effect of TAFEMgO NPs on the activated PT (prothrombin time) and the APTT (activated partial thromboplastin time). * Significance at *p* ≤ 0.005, ** at *p* ≤ 0.001 and *** at *p* ≤ 0.0001.

**Figure 8:**
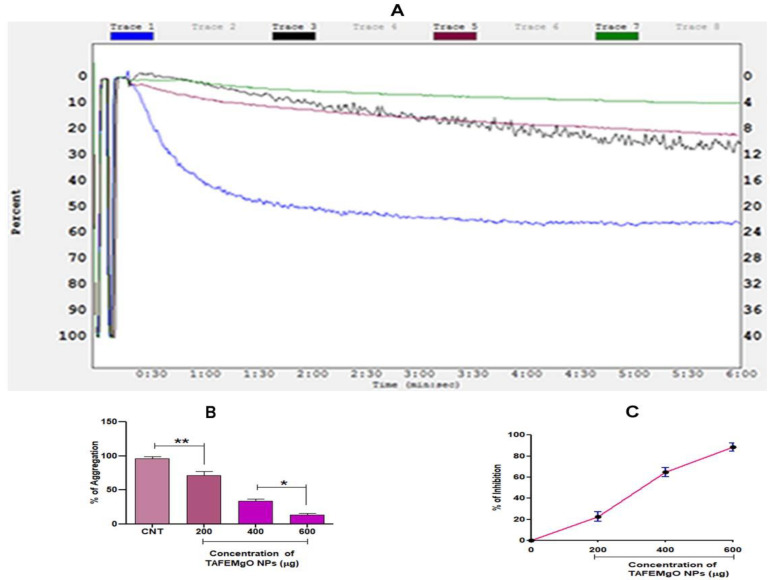
The impact of TAFEMgO NPs on ADP-agonist-induced platelet aggregation: (**A**) platelet aggregation traces: trace 1ADP 10 M; trace 2-ADP 10 M + 200 μg TAFEMgO NPs; trace 3ADP 10 M + 400 μg TAFEMgO NPs; and trace 4ADP 10 M + 600 μg TAFEMgO NPs. All the values represented were measured from three experiments and presented as mean ± SD. (**B**) percent platelet aggregation (%) in dose-dependent manner, (**C**) percent platelet aggregation inhibition (%) in dose-dependent manner. * Significance at *p* ≤ 0.005, ** at *p* ≤ 0.001.

To check the interference of TAFEMgO NPs with the blood coagulation cascade, prothrombin time, plasma recalcification time, activated partial thromboplastin time (in vitro) and mouse-tail-bleed time (in vivo) were performed. Interestingly, TAFEMgO NPs exhibited the anti-coagulant effect by delaying the time required for the clotting of the citrate-added plasma (885 s) from the control (193 s) plasma sample (Figure 7A). In addition, TAFEMgO NPs also showed the anticoagulant effect in mouse-tail-bleeding time by delaying the clotting time in the PBS-treated control from 106 s to 935 s at a dosage of 50 mg/kg (Figure 7B). Interestingly, TAFEMgO NPs also delayed the blood coagulation time of both APTT and the PT, suggesting an anticoagulant effect of TAFEMgO NPs. It might be because of its interaction with the factors in the common blood-clotting pathway (Figure 7C). In addition, TAFEMgO NPs was found to inhibit the platelet aggregation caused by agonist-ADP and the observed inhibition was 86.63% with an IC_50_ of 317.2 μg/μL (Figure 8).

### 2.8. TAFEMgO NPs Are Nontoxic Biogenic Molecules

**Figure 9:**
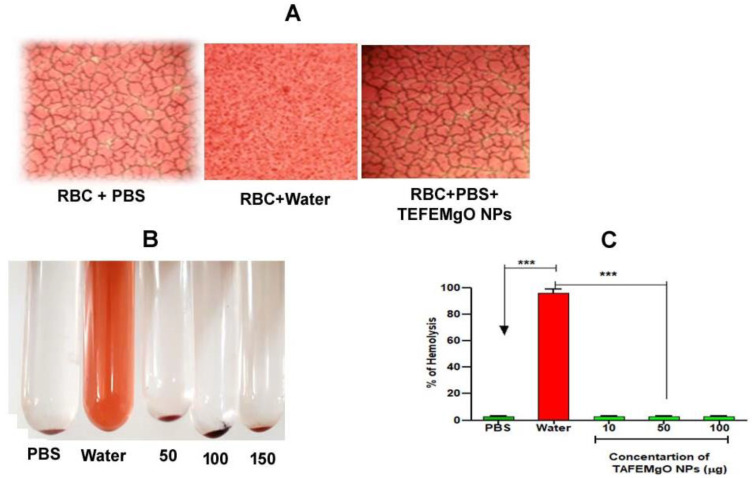
Direct hemolytic assay: (**A**) microscopic image of TAFEMgO NPs-protected RBC, (**B**) pictorial representation of TAFEMgO NPs depicting RBC hemolytic assay. (**C**) The % of hemolysis was determined at 540 nm as the measured amount of released Hb. *** Significance at *p* ≤ 0.0001.

TAFEMgO NPs did not induce hemolysis in the red blood cells and were not found to induce any hemorrhagic and paw-edema like toxic and inflammatory responses in the experimental mice (data not shown). This indicated the nontoxic nature of TAFEMgO NPs. Figure 9 indicates the effect of TAFEMgO NPs on red blood cells.

## 3. Discussion

Oxidative stress is the key elicitor of deadly lifestyle metabolic and physiological diseases, such as cancer, diabetes, hemolytic anemia, atherosclerosis, inflammatory conditions, high blood pressure, thrombosis, and cardiovascular and neurodegenerative diseases [16,17]. Therefore, the need for effective exogenous antioxidants derived from plant sources is gaining much attention as a therapeutic intervention for oxidative stress [18,19,20,21]. The metal oxide nanoparticles synthesized by physico-chemical strategic methods, like zinc oxide (ZnO), copper oxide (CuO), cobalt oxide (CoO), nickel oxide (NiO), and chromium oxide (Cr_2_O_3_), were found to cause potential carcinogenicity and environmental toxicity due to their high oxidizing and reducing power on exposure to hazardous reducing agents, such as organic solvents [22,23,24]. Therefore, their clinical and biological applications as therapeutic molecules are limited. The rationale of the current study is nanoparticles fabricated from physical and chemical methods are toxic to the cells. Green technology has been emerging as an alternative method as it involved phytochemical-coupled nanofabrication. Most importantly, micro size phytochemical encapsulation augments therapeutic potency by eliminating their toxic effect. Medicinal plant components were considered to be the key resources for green biosynthesis of functional nanoparticles due to enriched, stored phytochemicals. Perhaps phytochemicals impart therapeutic potency and eliminate the toxic effects of the metal nanoparticles by providing the unique physical, magnetic, thermal, optical, electrical, and chemical properties [25]. Therefore, several medicinal plants, such as *Lawsonia inermis*, *Artemisia anua* hairy root, *Amarantus blitum*, *Lime* peel extract, *Tragacanth gum*, *Piper nigrum*, *Limonia acidisima*, *Monascus purpureas*, *Moringa oleifera*, *Opuntia dileni haw Boswellia carteri resin*, and sugar cane juice were extensively used in the green synthesis technology [26,27]. Metal nanoparticles synthesized using green technology exhibited anticancer, antibacterial, antioxidant, and anti-inflammatory properties with the least toxicity [28,29]. *Terenna asiatica* plants have been extensively utilized in folk traditional medicine practices to treat several lifestyle and infectious diseases, but they are rarely validated. On the other hand, MgO nanoparticles were extensively studied for their antimicrobial potential. Therefore, MgO nanoparticles were fabricated using TAFE (*Terenna asiatica* fruit extracts) and evaluated their effects in vitro and in vivo on sodium nitrite and diclofenac-induced oxidative stress model systems.

The characterization data of TAFEMgO NPs revealed they possess an optical property, are spherical/polygonal in shape, and are in pure form [30,31]. TAFEMgO NPs exhibited DPPH-scavenging ability attributed to the conjugated functional groups from TAFE or the controlled precipitation of MgO nanoparticles.

RBCs perform multiple cellular functions, but they are often susceptible to oxidative stress. RBCs exposed to ROS produce high levels of Ca^2+^ through chemical signaling and allows them to enter the cells [32]. High concentrations of Ca^2+^ cause Ca^2+^-sensitive scrambling of erythrocyte membranes, leading to eryptosis or RBC death [33]. The RBC, after undergoing eryptosis by the ROS intern, produces RBC-mediated ROS, the key contributing factors for uncontrolled intravascular coagulation and vital organ damage. Therefore, RBC protection by the exogenous antioxidant plays a crucial role in regulation of oxidative induced damage and stress related many of lifestyle diseases.

TAFEMgO NPs restored the level of protein carbonyl content, lipid peroxidation, and the total thiol level in the red blood cells, revealing their usefulness in managing oxidative-stress-induced RBC damage. In addition, TAFEMgO NPs also mitigate DFC-induced in vivo oxidative damage in tissue (kidney, liver and small-intestine) homogenates by restoring the antioxidant status. Over the past decades, innumerable reports documented the antioxidant, antimicrobial, anticancer, and anti-inflammatory roles of green biosynthesized metal oxides biofunctional NPs, like, Mgo, TiO_2_, ZnO, CuO, SnO_2_, ZrO_2_, AgO, and CeO_2_ [34] However, Mina Sarani et al. in 2022 reported the potent cytotoxic effect of biofunctional green synthesized metal oxide nanoparticles, such as Bi_2_O_3_, Zn-doped Bi_2_O_3_ and Mn-doped Bi_2_O_3,_ from the *Salvadora persica* extracts act as the potential green functional reactives, act as reduction and capping agents [35]. Our study also suggests and infers that the activity of potential cellular enzymes with known antioxidant properties, such as SOD and CAT, decreased significantly in sodium nitrite and diclofenac-induced RBCs and in the rats’ vital organ homogenates, respectively. TAFEMgO NPs significantly stabilized the activity of SOD and CAT and mitigated vital organ damage against diclofenac-induced oxidative stress. This appears to be consistent with the normal results obtained from biochemical parameters and thus, partly verifies the safety of TAFEMgO NPs. In support of our findings, Zhang et al. documented the nontoxic property of silver nanoparticles as they showed no adverse effect on the rat’s liver [36,37,38]. The protective role of TAFEMgO NPs was also adjudged by measuring the biochemical parameters. Diclofenac toxicity decreased the levels of total serum proteins (albumin and globulin) and elevated the levels of total, direct, and indirect bilirubin. SGOT and SGPT are hepatic, enzymes, and their elevated levels in the circulatory system are an indication of liver damage [39,40]. The fundamental role of the ALP enzyme is the transfer of metabolites across the cell membrane, and the rise of this enzyme also suggests liver failure [41]. Interestingly. TAFEMgO NPs normalized all the biochemical parameters through their free-radical-quenching potential.

Furthermore, ROS and RBC-mediated ROS may alter hemostasis, either by activating platelets and coagulation factors leading to thrombosis [42]. Thrombosis is characterized by the formation of an unwanted clog/clot in the blood vessels of arteries and veins resulting in disease pathogenicity, which is the key cause of mortality and morbidity worldwide. Currently, anticoagulants and antiplatelet agents have been extensively used by medical practitioners despite their life-threatening side effects [43]. Thus, several research groups have been seeking to design an anticoagulant and antiplatelet agent with the least side effects. TAFEMgO NPs showed both anticoagulant and antiplatelet properties. TAFEMgO NPs enhanced the clotting time of both PT and APTT [44,45]. Therefore, the anticoagulant effect of TAFEMgO NPs could be due to the interference of both intrinsic and extrinsic pathways of blood coagulation. Hyper activation of platelets play a vital role in pathogenecity and physiology of thrombosis [46,47,48]. TAFEMgO NPs inhibited ADP-induced platelet aggregation, strengthening its observed therapeutic potential. Above all, TAFEMgO NPs did not cause any toxic effects, hemorrhage or inflammatory edema in mice in vivo models used in experiments and were unable to lyse erythrocytes, supporting their nontoxic nature imparted from the phytochemicals of the *Tarenna asiatica* fruit extract (TAFE).

## 4. Materials and Methods

### 4.1. Chemicals and Reagents

Magnesium nitrate (Mg(NO_3_)_2_6H_2_O), DPPH (1,1-diphenyl-2-picrylhydrazyl), NaNO_2_, EDTA, TEMED, 2,4-dinitrophenylhydrazine (DNPH), TCA, methanol, DTNB (5,5′-dithiobis-(2-nitrobenzoic acid)-Elman’s reagent, SDS, acetic acid, thiobarbituric acid, hydrogen peroxide (H_2_O_2_), APTT and PT reagents were used (Sigma Aldrich, Burlington, MA, United States). Blood samples were collected through vein punctures from healthy donors and were used for the isolation of 10% hematocrit and platelet rich plasma (PRP) and ADP.

### 4.2. Preparations of Terenna Asiatica Fruit Extract (TAFE)

*Terenna asiatica* fruits were collected from the forest area of Siddara Betta, Tumkur. They were washed thoroughly and dried for 48 h at room temperature. Fruits were powdered by mechanical homogenization using a pestle and mortar. The powder was weighed (0.6 g) and was added with a known amount of dH_2_O. The suspension was mixed and kept for stirring at 40 °C for 2 h. Then, the mixture was cooled and further filtered using Whatman no-1. The plant extracts were used as a solvent for green biosynthesis of the magnesium oxide (MgO) nanoparticle. Hence, the components in the extracts were predicted to act as a stabilizers and reducing agent in the formation of biofunctional TAFEMgO NPs.

### 4.3. Green Biosynthesis of TAFEMgO NPs

**Figure 10:**
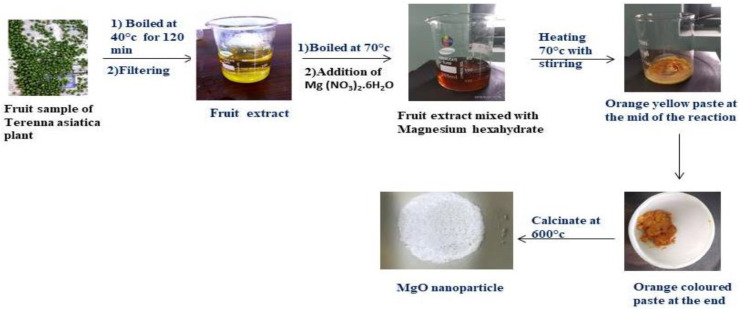
Extraction and processing scheme of Terenna asiatica plant fruit sample and preparation of biofunctionalized MgO nanoparticle.

To obtain a clear solution of TAFE, 40 mL of dH_2_O was added to 0.6 g of TAFE powder and mixed well by continuous stirring for 120 min at 40 °C. A total of 4 g of magnesium nitrate [Mg (NO_3_)_2_6H_2_O] was added to the TAFE solution, and mixed to kept in a sand bath with constant stirring for 4 h at 75 °C till it forms a brown color resin (Figure 10). The final product was calcinated for 8 h in a furnace at 600 °C to obtain a white powder of MgO [49].

### 4.4. Characterization of TAFEMgO NPs

TAFEMgO NPs were identified and characterized by employing various chemical and spectroscopic techniques, such as UV–Visible spectrophotometer (Jasco v-670 UV-VIS-NIR spectrometer, Tokyo, Japan) from the wavelength of 200 to 850 nm, energy-dispersive X-ray diffraction (JCM-6000PLUS, New Delhi, India), FTIR spectroscopy (Thermo Nicolet Is 50- Thermo Fisher, Waltham, MA, USA) using the wave number 400–4500 cm^−1^, scanning electron microscopy (JCM-6000PLUS, New Delhi, India), transmission electron microscope (Jeol/JEM 2100, JEOL, Peabody, MA, USA), selective area electron diffraction, and X-ray diffraction detector (Bruker D8 Advance XRD diffractometer, Bruker, Karlsruhe, Germany) using the standardized procedure.

### 4.5. Estimation of Antioxidant Property by DPPH Method

The antioxidant ability of TAFEMgO NPs was estimated, as described earlier by [50] using DPPH (2,2-diphenyl-1-picrylhydrazyl) assay. TAFEMgO NPs (0–100 μg) were taken in 50% methanol and made up to 2.5 mL and thoroughly mixed. Then, 0.14 mM DPPH (140 μL) reagent was added, and placed for 30 min in dark room. Optical density was taken at 517 nm against control having 50% methanol. The antioxidant property of TAFEMgO NPs was calculated as follows.

(1) $$\begin{array}{l} Percent\;\left(\%\right)Scavenging\;activity\\ =\frac{\left(\mathrm{Absorbance}\;\left(\mathrm{OD}\right)\;\mathrm{of}\;\mathrm{the}\;\mathrm{control}\;-\;\mathrm{Absorbance}\;\left(\mathrm{OD}\right)\;\mathrm{of}\;\mathrm{the}\;\mathrm{sample}\right)\times 100}{\mathrm{Absorbance}\;\mathrm{of}\;\mathrm{the}\;\mathrm{control}} \end{array}$$

### 4.6. Human Blood Collection and 2% Hematocrit Preparation

Human blood samples were collected from healthy individuals (aged 20 to 25 old) who were non-smokers, non-alcoholics, and did not take any medications. Acid citrate dextrose (ACD) anticoagulant was prepared using 85 mM sodium citrate, 71 mM citric acid, and 111mM dextrose. Then, freshly drawn blood was added to a mixture in ACD and centrifuged at 37 °C for 10 min at 800 rpm. RBCs were then washed three times using 10 mM PBS with pH 7.4, hematocrit (2%) was prepared using washed RBC with 10 mM PBS, and it was used for further assays.

### 4.7. Oxidative Stress-Induced by NaNO_2_

The method of [51] was followed to induce oxidative stress. Freshly prepared 1 mL of hematocrit (2%) was treated with 20 μL of NaNO_2_, and the different doses of TAFEMgO NPs (0–150 μg) were added and were kept for incubation at room temp. for 30 min. Following incubation time, the 2 mg/mL concentration of reaction mix was taken from the respective tubes to study the effect of TAFEMgO NPs on oxidative stress markers. NaNO_2_ alone treated with RBC was considered a reference control, and RBC alone without NaNO_2_ was a positive control.

### 4.8. Determination of Lipid Peroxidation (LPO)

The method of [52] was used to assess the LPO. In dry test tubes, NaNO_2_ was treated with 2 mg of protein from (2% hematocrit obtained from RBC lysate, and TAFEMgO NPs (0–100 μg) were added with 1.5 mL acetic acid (pH 3.5), 0.2 mL of 8% SDS, and 1.5 mL TBA (0.8%). The mixtures were subjected to incubation at 45 to 60 °C for 45 min and then, 3 mL of 1-butanol was added, the resultant TBARS (TBA reactive substance) mixture was subjected for centrifugation at 5000 rpm for 15 min, and the intensity of the samples were determined photometrically at 532nm.

### 4.9. Estimation of Protein Carbonyl Content (PCC)

Method [53] followed to measure the PC content in the samples using DNPH. NaNO_2_ (10 mM) and TAFEMgO NPs (0–100 μg) were used to treat 1 mL of lysate of RBCs (2 mg protein/mL). These mixtures were kept for incubation for 1 h after adding 5 μL of 10mM DNPH in 2N HCl with recurrent shaking. Only 2N HCl was added to control tubes.. Following incubation time, the reaction mixtures were precipitated with TCA 20%, further subjected to centrifugation at 5000 rpm for 15 min, and then the obtained precipitates were washed thoroughly with acetone before being resolubalized in 1 mL of 20 mM Tris-buffer (pH 7.4), added with 2% SDS and 0.14M NaCl. The solubalized samples were centrifuged at 360 rpm for 15 min. Then, absorbance of the resultant protein solutions were calculated using a molar absorptivity of 22 mM and was reported as mol carbonyl groups/mg protein.

### 4.10. Estimation of Total Thiol (TT)

To evaluate the total thiols, the described method [54] was followed. Briefly, NaNO_2_ (10 mM) and TAFEMgO NPs (0–100 μg) were added to 1 mL of RBC lysate (2 mg protein/mL). An amount of 0.375 mL of 0.2M Tris-HCl buffer (pH 8.2) added with 10 mM di-thiol-bis-nitro benzoic acid (DTNB) and 1.975 mL of methanol were vertexed and incubated for 30 min. The tubes were centrifuged for 10 min at 5000 rpm. The clear supernatants of the samples were collected and further measured for photometric absorbance at 412 nm, and the thiol content was represented as n mol of DTNB oxidized/mg protein.

### 4.11. Determination of Activities of CAT and SOD 

The reported procedures of [55,56] were followed. Briefly, 10 mM NaNO_2_ was used with RBCs (2% hematocrit), the TAFEMgO NPs (0–100 μg) were incubated for 2 h, and then using distilled water, the erythrocytes were lysed. The lysate was collected and utilized for the assay of SOD and CAT. For the SOD activity, 1 mL of a reaction mixture with 16mM phosphate-buffer (pH 7.8), containing 8 mM/0.8 mM of TEMED/EDTA, respectively, were used for the assay. To that, 0.1 mL of RBCs lysate (0.05 mg proteins) was added to reaction mixture, and then, the OD value was measured for 1 min at 406nm. Similarly for CAT assay, 1mL of the RBC lysate (0.05 mg proteins) was taken in 100mM phosphate-buffer (pH 7.4) containing 8.8 mM H_2_O_2_. The CAT activity was measured as rate of H_2_O_2_ decomposition/min/mg protein and was calculated in terms of the reduction in the OD value at 240 nm for 3 min.

### 4.12. Animal Grouping and Sample Dose Administration

Male Sprague Dawley rats were chosen as the animal model for the in vivo studies. They were obtained from the Institution of Liveon Bio labs in Tumkur, Karnataka, India and housed in polypropylene cages (six rats/cage). The rats for the experimental studies selected weighed around 75–100 g and were about six to eight weeks old. The experimental animals were fed with recommended standard laboratory diet in the form of pellets and were provided free access to water. In all the cages, the temperature (25 ± 3 °C), the humidity of 55–65%, with automated of 12 h light/12 h dark light cycles were maintained. The Institutional Animal Ethics Committee approved the experiment protocol and study design, and approval was given under the number LBPL-IAEC-47-05/19. The male Sprague Dawley (75–100 g) rats were grouped into seven and comprised of six rats in each group, and the protocol was followed as below:
Group IControl (normal saline).Group IIDiclofenac alone.Group IIISilymarin (25 mg/kg body weight/day) was injected intraperitonially, and after 45 min diclofenac (50 mg/kg body weight/day) was administered.Group IVTAFEMgO NPs (50 mg/kg body weight/day) was injected intraperitonially, and after 45 min diclofenac (50 mg/kg body weight/day) was administered.Group VTAFEMgO NPs (75 mg/kg body weight/day) was injected intraperitonially, and after 45 min diclofenac (50 mg/kg body weight/day) was administered.Group VITAFEMgO NPs (100 mg/kg body weight/day) was injected intraperitonially, and after 45 min diclofenac (50 mg/kg body weight/day) was administered.Group VIITAFEMgO NPs (100 mg/kg body weight/day) alone administered.

Rats were given treatment for seven days, and after the last dose, they were made to fast without food for 12 h. Then on eighth day, animals were anesthetized with diethyl ether, the animals were euthanized, and blood samples were obtained through heart perforations. For the analysis of biochemical parameters, blood samples (2 mL) were collected from the aorta using sterile voiles without anticoagulant. The biochemical parameters, such as total serum protein, albumin, globulin, total bilirubin (direct and indirect levels), SGOT, SGPT, and alkaline phosphatase were analyzed. The small intestine, kidney, and liver tissues were removed from the experimental animals and preserved in phosphate buffer saline solution before being homogenized for biochemical examination. The organs were also stored in 10% formalin for histopathological analysis.

### 4.13. Histopathological Examination

For histopathological examination, the liver, kidney, and small intestine tissues from all the groups were processed and embedded in paraffin. Hematoxylin and eosin stains were used to stain sections of 3–5 μm thickness.

### 4.14. Determination of In Vivo Antioxidant Activities

The experimental rats’ vital organs (livers, kidneys, and small intestines) were collected using chilled ice-cold 0.1 M PBS, the tissue samples were homogenized thoroughly, and the samples were used for the analysis of stress markers.

### 4.15. Plasma Recalcification Time

The method of [57] was employed. TAFEMgO NPs (0–160 μg/μL) were pre-incubated for 1 min with human platelet rich plasma (0.2 mL) in the presence of 10mM Tris-HCl buffer (20 μL) of pH 7.4. The plasma samples were further subjected for determination of clotting time after the mixtures pre-incubated with 20 μL of 0.25 M CaCl_2_.

### 4.16. Prediction of Prothrombin Time and Thromboplastin Time 

Prediction of activated thromboplastin time and prothrombin time were performed, as described by [58]. Citrated human plasma (100 μL) and TAFEMgO NPs (0–120 μg) were pre-incubated for a minute. For the activated PTT test, about 100 μL of Liquicelin-E phospholipids reagent was preactivated at 37 °C for 3-4 min. The coagulation process was initiated by addition of 0.02 M CaCl_2_ (100 μL). For the PT test, about 200 μL of rabbit brain thromboplastin (UNIPLASTIN) was mixed with the plasma to initiate the clot. In both, the test time for clot appearance against the given light source was recorded. The activated PTT ratio in comparison with international normalized ratio (INR) for the prothrombin time was calculated at each point against the suitable control.

### 4.17. Bleeding Time

The method of [59] was employed. TAFEMgO NPs (0–120 μg) in 30 μL of 10 mM PBS were administered into mice tail veins and allowed to stand for 10 min. After, mice were anesthetized by diethyl ether inhalation, then sharp cuts about 3 mm long were made at the tail tips of the mice. The tails were dipped vertically in phosphate buffered saline (PBS) that had been pre warmed at 37 °C and the bleeding times of the control and tested groups were recorded.

### 4.18. Determination of Platelet Aggregation

Platelet aggregation was carried out, as previously explained by [60]. The Chronolog Dual-Channel blood/Optics-Lumi Aggregation system was used for the study. Platelet rich plasma (0.25 mL) aliquots were pre-warmed with different doses of TAFEMgO NPs (200–600 μg). The aggregation of the platelets was started independently, and after the addition of the agonist ADP, the aggregation was monitored for 6 min and recorded.

### 4.19. Determination of Direct Hemolytic Property

The direct hemolytic activity was measured, as previously described by [61]. About 1 mL of human red blood cells were added with 9 mL of 10 mM PBS (pH 7.4). 1 mL of this diluted blood samples were further subjected for incubation for 1h at 37 °C with different concentrations of TAFEMgO NPs (0–150 μg). The hemolytic reaction was stopped with addition of 9 mL ice-cooled 10 mM PBS (pH 7.4). The samples were centrifuged at 1500 rpm for 10 min at 37 °C. The amount of released Hbin the supernatant of samples were measured and compared at 540 nm to determine the level of hemolysis.

### 4.20. Statistical Analysis

The collected data of the results were analysed statistically and reported as mean±SD. Using one-way ANOVA, individual parameters were compared using Prism Pad software (Version 5.0) (Graph Pad Software, Inc., San Diego, CA, USA). *p*-value (*p* ≤ 0.05) were appropriately considered as significant.

## 5. Conclusions

*Terenna asiatica* fruit possess the medicinal qualities attributed to phytoconstituents, such as alkaloids, tannins, flavonoids, and glycosides, and are the key mediators of biosynthesized MgO functional nanoparticles. The TAFEMgO NPs were further characterized to confirm the biosynthesis by employing methods and techniques, like SEM, XRD, TEM, FTIR, EDX, and UV spectroscopy.

The data confirmed the Mgo NPs had average particle sizes with 66.83 nm, and they are spherical in morphology and devoid of contamination. The TAFEMgO NPs potentially scavenged the DPPH free radicals, and through their antioxidant properties, they protect the sodium nitrite and dicolofenac-induced oxidative stress in RBCs and vital organs (small intestine, liver, and kidneys) by regulating the stress markers. In addition, TAFEMgO NPs showed anticoagulant and antiplatelet activities without toxic effects. Hence, TAFEMgO NPs are potential contenders in the regulation and management of oxidative-stress-mediated metabolic and inflammatory diseases. Understanding the molecular mechanism of TAFEMgO NPs on oxidative stress and thrombosis is of great interest and may provide the future directions for the research and development of effective treatment strategies using green synthesized biofunctional metal oxide nanoparticles.

## Figures and Tables

**Table 1:** Effect of TAFEMgO NPs on biochemical parameters in Sprague Dawley (75–100 g) male rats.

Biochemical Parameter	Group I Control	Group II DFC Alone	Group III DFC+Syl	Group IV DFC + TAFEMgONPs (50 mg/kg)	Group V DFC + TAFEMgONPs (75 mg/kg)	Group VI DFC + TAFEMgONPs (100 mg/kg)	Group VII TAFEMgONPs (100 mg/kg)
Albumin (g/dL)	3.92	1.365	3.34	3.55	3.20	3.083	3.29
Globulin (g/dL)	4.48	2.73	4.26	4.06	4.49	4.683	4.71
Total protein (g/dL)	8.4	5.095	7.6	7.7	7.70	8.1	8.0
Bilirubin (total) (g/dL)	0.27	0.605	0.26	0.32	0.323	0.263	0.3
Bilirubin (Direct) (g/dL)	0.08	0.3	0.04	0.11	0.12	0.07	0.096
Bilirubin (Indirect) (g/dL)	0.19	0.305	0.22	0.2	0.203	0.193	0.203
SGOT (U/L)	176.4	218.0	180.6	165.8	167.23	174.3	176.06
SGPT (U/L)	164.5	245.6	178.6	156.1	159.4	160.13	157.26
Alkaline phosphatase (U/L)	115.6	200.0	149.6	131.23	120.23	123.6	109.14

Note: All samples were administered as mg/kg body weight/day.

## Data Availability

Not applicable.

## Supplementary Materials

The following supporting information can be downloaded at: https://www.mdpi.com/article/10.3390/molecules27165162/s1. Figure S1—Super oxide dismutase (SOD) activity: Prior to treatment with NaNO_2_ (10 mM), RBCs were pre-incubated for 10 min with various doses (25–100 g/mL) of TAFEMgO NPs at 37 °C. Figure S2—Catalase (CAT) activity: Prior to treatment with NaNO_2_ (10 mM), RBCs were pre-incubated for 10 min with various doses (25–100 g/mL) of TAFEMgO NPs at 37 °C. Figure S3—Effect of TAFEMgO NPs on diclofenac-induced oxidative stress in the liver, Kidney, small intestine. Figure S4—Effect of TAFEMgO NPs on diclofenac-induced oxidative stress in the liver, Kidney, small intestine.

## Sample Availability

Samples of the compounds TAFEMgONPs are available from the authors.

## Abbreviations

MgO NPs—magnesium oxide nanoparticles, ROS—reactive oxygen species, RBC—red blood cells, DPPH—di-phenyl-2-picryl-hdrazyl, DNPH—di-nitro-phenyl-hydrazine, TCA—trichloroacetic acid, DTNB—5,5′-dithiobis-2-nitrobenzoic acid, SDS—sodium dodecyl sulfate, TEMED—tetra methyl ethylene diamine, EDTA—ethylene diamine tetra acetic acid, APTT—activated partial thromboplastin time, PT—prothrombin time, PRP—platelet rich plasma, ADP—adenosine di-phosphate, TAFE—*Terenna asiatica* fruit extract, XRD—X-ray diffraction detector, TEM—transmission electron microscope, SAED—selected area (electron) diffraction, SEM—scanning electron microscopy, EDX—energy dispersive X-ray diffraction, FTIR—Fourier transform infrared spectrophotometry, DFC—diclofenac.
