# Supplementary material for: Effect of Biofunctional Green Synthesized MgO-Nanoparticles on Oxidative-Stress-Induced Tissue Damage and Thrombosis
Source: Molecules. 2022 Aug 12;27(16):5162. doi: 10.3390/molecules27165162 (PMC9413574; doi:10.3390/molecules27165162)
Supplement: Supplementary file 1 [file molecules-27-05162-s001.zip › molecules-1813637-supplementary.pdf]

Supplementary data

S1

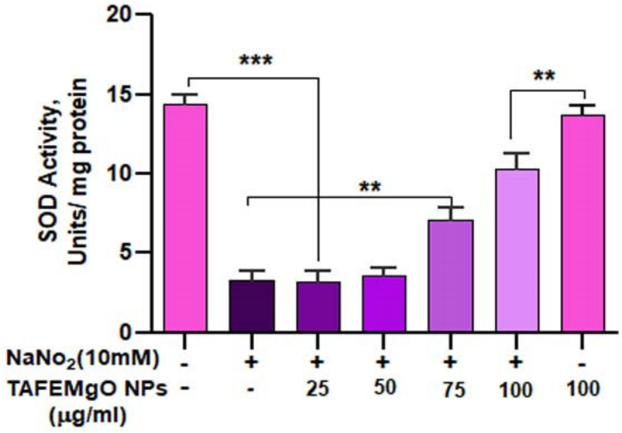

Figure S1. Super oxide dismutase (SOD) activity: Prior to treatment with $NaNO_2$ (10 mM), RBCs were pre-incubated for 10 min with various doses (25-100 g/mL) of TAFEMgO NPs at 37 °C. The results are expressed in average enzyme units per mg protein and are presented as mean SEM (n = 3).

S2

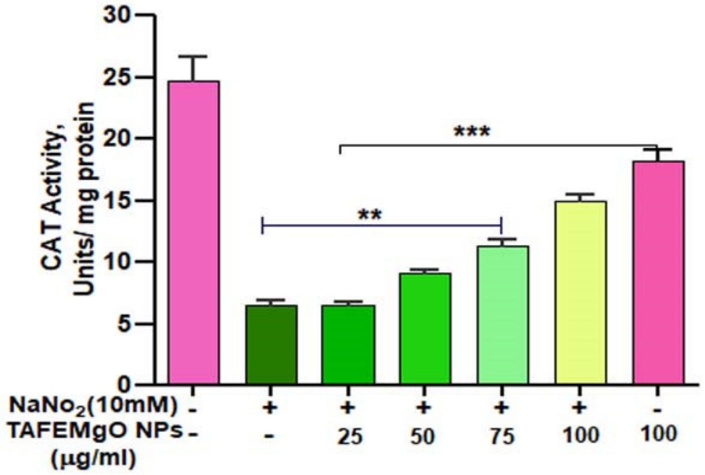

Figure S2. Catalase (CAT) activity: Prior to treatment with $NaNO_2$ (10 mM), RBCs were pre-incubated for 10 min with various doses (25-100 g/mL) of TAFEMgO NPs at 37 °C. The results are expressed in average enzyme units per mg protein and are presented as mean SEM (n = 3).

S3

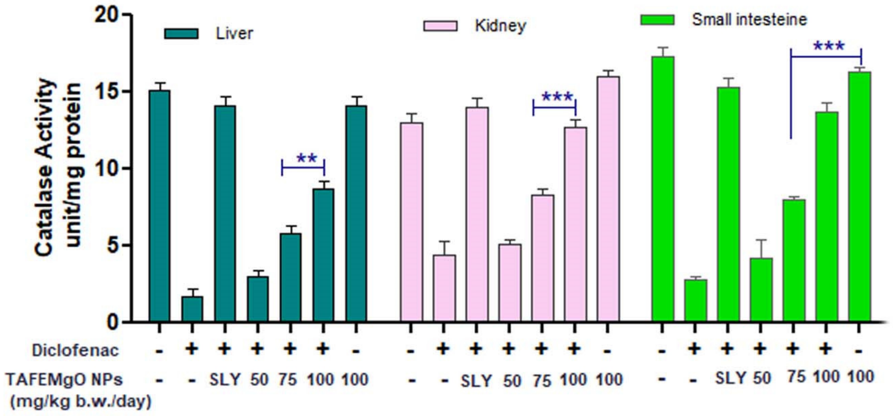

Figure S3. Effect of TAFEMgO NPs on diclofenac-induced oxidative stress in the liver, Kidney, small intestine: Superoxide dismutase: Control, Diclofenac (50 mg/kg b.w./day), SLY + Diclofenac (25 mg/kg b.w./day) TAFEMgO NPs + Diclofenac (50 mg/kg b.w./day) TAFEMgO NPs + Diclofenac (75 mg/kg b.w./day) TAFEMgO NPs + Diclofenac (100 mg/kg b.w./day) and TAFEMgO NPs alone (100 mg/kg b.w./day). In comparison to the toxicity control group, the data is presented as mean (n = 3) SEM. To represent the data, SEM mean (n = 3) was used**.**

S4

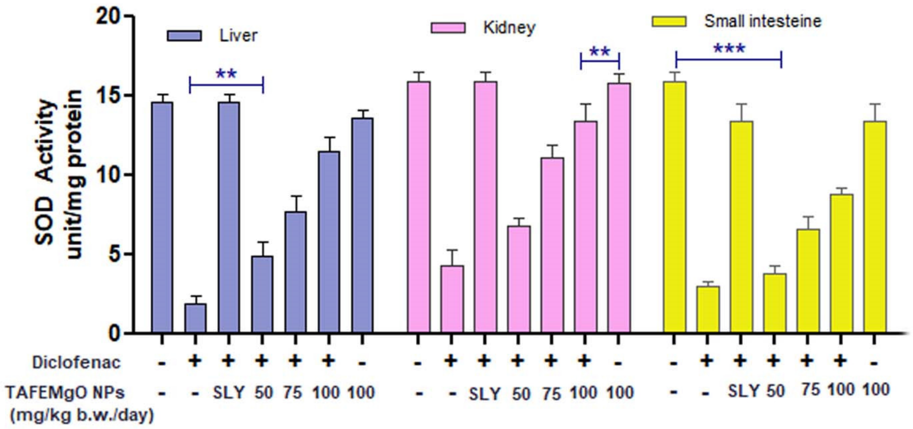

Figure S4. Effect of TAFEMgO NPs on diclofenac-induced oxidative stress in the liver, Kidney, small intestine: Catalase: Control, Diclofenac (50 mg/kg b.w./day), SLY + Diclofenac (25 mg/kg b.w./day) TAFEMgO NPs + Diclofenac (50 mg/kg b.w./day) TAFEMgO NPs + Diclofenac (75 mg/kg b.w./day) TAFEMgO NPs + Diclofenac (100 mg/kg b.w./day) and TAFEMgO NPs alone (100 mg/kg b.w./day). In comparison to the toxicity control group, the data is presented as mean (n = 3) SEM. To represent the data, SEM mean (n = 3) was used**.**
